# Genomics of chromophobe renal cell carcinoma: implications from a rare tumor for pan-cancer studies

**DOI:** 10.18632/oncoscience.130

**Published:** 2015-02-20

**Authors:** Kimryn W. Rathmell, Fengju Chen, Chad J. Creighton

**Affiliations:** ^1^ The Dan L. Duncan Cancer Center Division of Biostatistics, Houston, TX, USA; ^2^ Department of Medicine, Baylor College of Medicine, Houston, TX, USA; ^3^ Department of Bioinformatics and Computational Biology, The University of Texas M. D. Anderson Cancer Center, Houston, TX, USA; ^4^ Department of Urology, University of North Carolina, Chapel Hill, NC; ^5^ Department of Genetics, University of North Carolina, Chapel Hill, NC; ^6^ Department of Medicine, Division of Hematology and Oncology, University of North Carolina, Chapel Hill, NC

**Keywords:** ChRCC, chromophobe, kidney cancer, genomics, TERT, TCGA, mitochondria

## Abstract

Chromophobe Renal Cell Carcinoma (ChRCC) is a rare subtype of the renal cell carcinomas, a heterogenous group of cancers arising from the nephron. Recently, The Cancer Genome Atlas (TCGA) profiled this understudied disease using multiple data platforms, including whole exome sequencing, whole genome sequencing (WGS), and mitochondrial DNA (mtDNA) sequencing. The insights gained from this study would have implications for other types of kidney cancer as well as for cancer biology in general. Global molecular patterns in ChRCC provided clues as to this cancer's cell of origin, which is distinct from that of the other renal cell carcinomas, illustrating an approach that might be applied towards elucidating the cell of origin of other cancer types. MtDNA sequencing revealed loss-of-function mutations in NADH dehydrogenase subunits, highlighting the role of deregulated metabolism in this and other cancers. Analysis of WGS data led to the discovery of recurrent genomic rearrangements involving *TERT* promoter region, which were associated with very high expression levels of *TERT*, pointing to a potential mechanism for *TERT* deregulation that might be found in other cancers. WGS data, generated by large scale efforts such as TCGA and the International Cancer Genomics Consortium (ICGC), could be more extensively mined across various cancer types, to uncover structural variants, mtDNA mutations, themes of tumor metabolic properties, as well as noncoding point mutations. TCGA's data on ChRCC should continue to serve as a resource for future pan-cancer as well as kidney cancer studies, and highlight the value of investigations into rare tumor types to globally inform principals of cancer biology.

## INTRODUCTION

Chromophobe kidney cancer (ChRCC) is a rare subtype of renal cell carcinoma (RCC), a heterogenous group of cancers arising from the kidney nephron. As part of an effort by The Cancer Genome Atlas (TCGA), we comprehensively profiled 66 ChRCC cases at the molecular level [1]. This effort represented the first of a series of Rare Tumor Projects initiated by TCGA, where each project comprehensively profiles a rare and understudied cancer subtype (other rare tumors currently under study including adrenocortical carcinoma, cholangiocarcinoma, paraganglioma and pheochromocytoma, sarcoma, thymoma, testicular germ cell tumors, uterine carcinoma, and uveal melanoma). Molecular data platforms involved in our study of ChRCC included whole exome sequencing, whole genome sequencing, mitochondrial DNA (mtDNA) sequencing, DNA methylation arrays, and mRNA and microRNA sequencing. This review revisits key findings made in this study, and further considers their implications for the study of other cancers.

Our study of ChRCC was unique in many respects. The multi-platform molecular data generated for this understudied disease, with the associated analysis work, was carried out at a level that would never have been possible from smaller scale studies. Even when compared to recent comprehensive, multi-platform studies of other cancers (from TCGA and others), ours represented the first such study featuring both a large number of both high-coverage whole genomes (n=50 cases, sequenced at a coverage of 60X read depth) and mtDNA sequences (by Long-range polymerase chain reaction or LR-PCR), where both data types were integrated with data from the other platforms. These data should help enable new thinking in areas relevant to many distinct, and perhaps more common cancers beyond kidney chromophobe. In particular, our findings pointed to a truly novel mechanism of *TERT* up-regulation in cancer (differing substantially from that of the activating point mutations reported elsewhere [2, 3]) and raise some provocative questions, regarding the precise role of mtDNA mutations, in cancers utilizing oxidative phosphorylation. Our study demonstrates that large scale molecular profiling of an understudied cancer can reveal novel cancer mechanisms, and can provide insights into the biology of even more common cancers.

### Chromophobe renal cell carcinoma (ChRCC) is a distinct disease

ChRCC is one of the renal cell carcinomas, a heterogenous group of cancers arising from the kidney nephron. ChRCC is a rare tumor type accounting for approximately 5% of RCC cases [4]. ChRCCs, characterized by a highly specific karyotype, exhibit an indolent pattern of local growth, with greater than 90% ten-year cancer-specific survival for localized disease [5, 6], but aggressive features and metastasis can occur. While chromophobe kidney cancer is associated with multiple cytogenetic abnormalities [7], detailed evaluation of the somatic genetics of this cancer had not been performed previously. ChRCC is seen at high frequency in Birt-Hogg-Dubé (BHD) syndrome, an autosomal dominant cancer predisposition syndrome due to mutations in *FLCN*, as nearly 40% of BHD-associated kidney tumors are characterized as ChRCC. However, *FLCN* mutations are rarely observed in sporadic ChRCC [8-10]. ChRCC has also recently been reported in Cowden syndrome, which is associated with *PTEN* mutations [11].

Only in the past two decades has it has been recognized that RCC represents a collection of highly distinct tumors, with distinct molecular and genetic features potentially reflecting the cell-of-origin as well as independent processes of tumorigenesis. Distinct molecular signatures may be seen in different cancer types, and it is not uncommon for cancers arising in different organs to have greater similarity than those belonging to a classic pathologic subtype within a single organ [12, 13]. ChRCC was first described in 1985 as distinct from ccRCC because of unique morphologic features, including abundant cytoplasmic single-membrane vesicular structures that may arise from budding of the mitochondrial membrane [14, 15]. An eosinophilic variant of ChRCC was subsequently identified with abundant mitochondria, resulting in the characteristic granular eosinophilic cytoplasmic staining, with few vesicular structures.

Our multi-platform analyses clearly confirmed that ChRCC is a disease entity distinct from the more common RCC, clear cell renal cell carcinoma (ccRCC). The molecular differences between ChRCC and ccRCC are easily highlighted by their characteristic features. For example, ChRCC lack the mutational events involving *VHL* and chromatin remodeling genes on chromosome 3p that occur in the vast majority of ccRCC [16]. In contrast, ChRCC cases demonstrated a much larger percentage of *TP53* mutations (32% of the 66 cases) than are seen in ccRCC. ChRCC is characterized in part by a great degree of uniform chromosomal copy number alterations, with the majority of cases having loss of one copy of the entire chromosome, for most or all of chromosomes 1, 2, 6, 10, 13, and 17, while in ccRCC only the loss of chromosome 3p is seen to a similar degree. Interestingly, a fraction of the both ccRCC and ChRCC (~20%) show alterations impacting the PTEN/MTOR pathway with the greatest number of mutations in ChRCC being present within the *PTEN* gene. Thus, the PTEN/MTOR pathway could represent a therapeutic target in at least some of these cancers that could be shared by both ChRCC and CCRCC tumors, though one important caveat here is that even the presence of the same gene mutations may have differing effects dependent upon the cellular background.

### Likely cell of origin of ChRCC

As TCGA had previously carried out a comprehensive study of ccRCC [16] prior to our ChRCC study, we were able to compare the molecular profiles of these two types of RCC. From the perspective of multiple data platforms (copy number, whole exome, RNA sequencing, and DNA methylation), ChRCC appeared entirely distinct from ccRCC, which suggested that the two may arise from different cells of origin. In particular, we observed widespread differences in DNA methylation between ChRCC and ccRCC, involving over tens of thousands of genomic loci. In principle, some of these differences could involve cancer-relevant pathways, but other differences might also reflect the respective cells of origin of the two cancers [17]. Previously, ChRCC had been postulated to arise from intercalated cells in the distal convoluted tubule of the kidney nephron, while ccRCC could arise from cells in the proximal convoluted tubule. This theory is based on previous work focusing on tubule-specific protein markers, by immunohistochemistry [18], but we sought in our study to revisit this question, using a global analysis of gene expression.

In addressing the question of the cell of origin of ChRCC, we used a creative analytical approach, involving an external expression profiling dataset from Cheval *et al*. [19]. In the Cheval study, normal tissue, from both mouse and human nephron specimens, was carefully microdissected from the glomerulus and seven anatomically defined nephron segments and subjected to gene expression profiling. The Cheval study associated both global gene expression patterns and individual gene markers with specific sections of the nephron, and-- to the benefit of future science--made the entire dataset publicly available, allowing us to analyze these data in the context of ChRCC. The actual analysis involving the TCGA and Cheval datasets was straightforward: For each gene in our TCGA kidney cancer dataset (combined ChRCC and ccRCC), expression values were centered across sample profiles (using the mean centroid of the two cancers); in a similar manner, within each of the human and mouse datasets from the Cheval study, values were centered across sample profiles. The respective centered datasets represented differential expression profiles, with essentially unitless values. For each TCGA and Cheval differential expression profile, we computed the global inter-profile correlation, using all ~4000 genes in common.

Our supervised analysis, globally comparing each TCGA ChRCC or ccRCC tumor expression profile to that of each sample in the Cheval nephron atlas, showed high mRNA expression correlations for ChRCC with distal regions of the nephron; ccRCC tumors demonstrated gene expression profiles, on the other hand, which correlated with patterns associated with the proximal nephron. These associations were strikingly clear, being observed in both the mouse and human nephron datasets. While our analysis associated ChRCC and ccRCC with a respective site of origin within the nephron, and not necessarily a specific cell of origin, when the previous immunohistochemistry studies of RCC are also taken into account [18], the best explanation to date for all of these data is that ChRCC and ccRCC do in fact have different cells of origin, as originally postulated. With this in mind, it is possible that therapies evolved for use on ccRCC tumors may not be successful in ChRCC.

Cancer cell of origin is a question relevant to many other cancers in addition to ChRCC. Knowledge of the cell of origin may provide insights into disease etiology, for example. Widespread molecular heterogeneity may be observed within other cancers sharing a common tissue-of-origin [20, 21]. In our study of RCC, we were able to put much of the observed molecular differences into some meaningful context, where the global gene expression differences had been previously noted [22] but without an overall framework to explain them. For our analytical approach to be applied to other cancers, a suitable molecular profiling dataset representing the candidate cells of origin would be needed, though in most cases, a suitable dataset may not be readily available. Recently, expression profiling data representing hundreds of cell types was made available by the FANTOM consortium [23], which data might be utilized in the analysis of other cancers, taking a similar approach to that of our use of the Cheval nephron dataset. In addition, as DNA methylation profiles of normal cell types become available in the future, such data might also be analyzed in the context of cancer.

### Metabolism and mitochondrial function in ChRCC

For some time, kidney cancer has been viewed as essentially a metabolic disease [24-26]. Mutations in a number of kidney cancer-associated genes, including *VHL*, *MET*, *FLCN*, *TSC1*, *TSC2*, *FH*, and *SDH*, result in dysregulation of metabolic pathways involved in oxygen, iron, energy or nutrient sensing. For example, germline mutations of *FH*, which lead to a metabolic shift to aerobic glycolysis, are associated with the development of the genetic syndrome of hereditary leiomyomatosis and renal cell cancer (HLRCC), which is characterized by an aggressive papillary type II renal cell carcinoma [27]. As another example, ccRCC is closely associated with *VHL* gene alterations that lead to stabilization and resulting accumulation of HIF-1α and HIF-2α; the increased HIF levels lead to both alterations in glutamine metabolism, and sensitivity to glutamine deprivation [28], as well as increased transcription of a number of downstream genes such as *GLUT1*, which enables transport of glucose for ATP production [24]. In TCGA's comprehensive molecular analysis of ccRCC, widespread molecular changes were associated with tumors having a poorer outcome; these changes implicated a metabolic shift, with tumors altering their usage of key pathways and metabolites in a “Warburg-like” effect. Worse survival in ccRCC correlated with up-regulation of pentose phosphate pathway genes and fatty acid synthesis genes, while better survival correlated with up-regulation of AMPK complex genes, multiple Krebs cycle genes, and PI3K pathway inhibitors (e.g. *PTEN*, *TSC2*) [16].

In direct contrast to ccRCC, previous studies using F-18-fluorodeoxyglucose PET/CT have suggested that ChRCC exhibits a non-glycolytic metabolic profile [29]. In addition, our ChRCC cases showed increased expression of genes involved in the Krebs cycle and the electron transport chain for generation of ATP[1]. Given the indicated prevalent role of mitochondria and metabolism in ChRCC, we sequenced mtDNA from 61 of our 66 ChRCC cases, using an LR-PCR-based amplification approach. MtDNA gene mutations in ChRCC all involved complex I of the electron transport chain, in particular in the NADH dehydrogenase 5 gene (*MT-ND5,* in 6 of our 61 cases); due to the type of mutations observed, these would be likely to result in loss of complex I activity. Furthermore, *MT-ND5*-mutated ChRCC cases were significantly associated with eosinophilic histology, which itself was associated with a phenotype of fewer copy number alterations and distinctive gene expression patterns.

Further studies to dissect the precise role of mitochondria DNA alterations could shed light on how core metabolic pathways may be altered in ChRCC and other diseases. Renal oncocytoma, a benign renal tumor that may also arise from the distal nephron, shares several similarities with ChRCC, including abundant, eosinophilic cytoplasm and densely packed mitochondria[15, 30]. It is believed that oncocytomas and ChRCC represent two ends of a tumor spectrum that also includes BHD-associated hybrid-oncocytic tumors, all of which have been found to have high levels of expression of mitochondrial and oxidative phosphorylation genes and to cluster together based on this feature [31]. Mitochondrial accumulation in renal oncocytomas was initially hypothesized as a compensatory mechanism of inefficient oxidative phosphorylation [32]. More recently, isolated loss of complex I activity was identified and determined to result from homoplasmic, somatically acquired mutations in mitochondrial complex I genes [32-34]. Our findings and that of other studies would suggest an irregular metabolic program supporting the growth of ChRCC [1, 29], one counter to the Warburg phenomenon observed commonly in ccRCC and many other cancers. However, elsewhere mutations in *MT-ND5* and other complex I genes have been hypothesized to lead to inactivation of oxidative phosphorylation and reliance on glycolysis [35]. Some functional studies into the role *MT-ND5* mutations have been carried out to date, including one by Hofhaus and Attardi [36], where loss of *MT-ND5* and complex I activity was found to occur in cell lines that developed resistance to an inhibitor of oxidative phosphorylation, and another by Park *et al*.[37], where *MT-ND5* mutations were associated with alteration of reactive oxygen species generation and apoptosis as well as with loss of oxidative phosphorylation.

**Figure 1:**
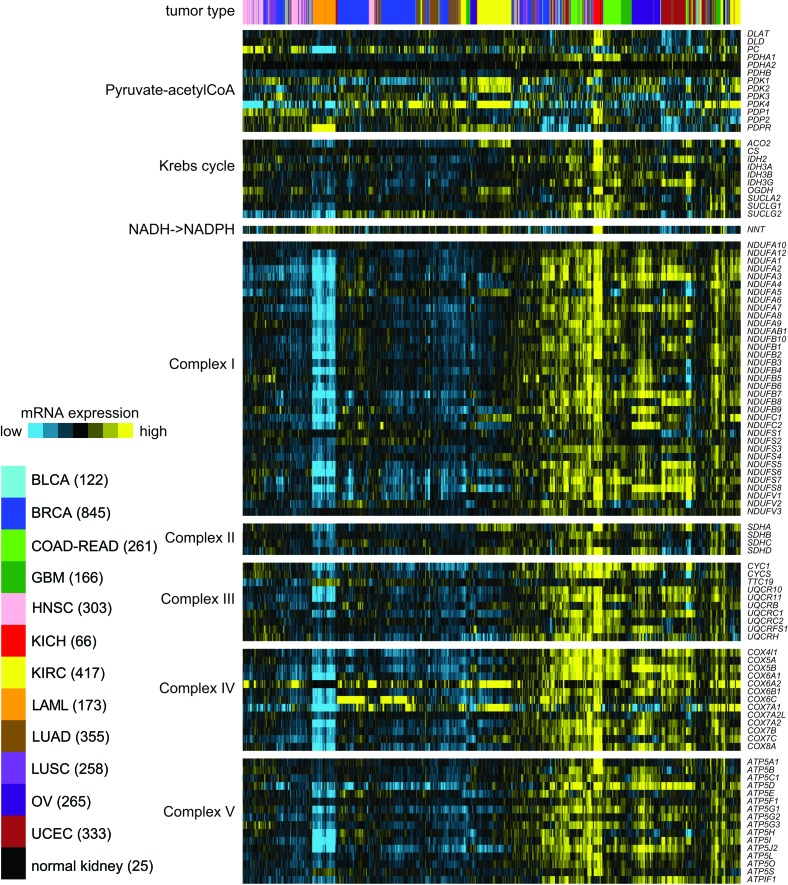
In a panel of human cancers of various tissues of origin (from TCGA, n = 3,564 samples), differential expression (relative to sample median) of genes related to Krebs cycle and Electron Transport Chain (ETC) RNA-seq profiles are from the recent TCGA pan-cancer multiplatform subtyping analysis, with the addition of sample profiles of ChRCC (KICH) and normal kidney from TCGA ChRCC study [1]. On the basis of the expression patterns of the selected genes, samples were clustered by unsupervised hierarchical method [50]. KICH, chromophobe renal cell carcinoma (ChRCC); BLCA, bladder cancer; BRCA, breast cancer; COAD, colon cancer; GBM, glioblastoma; HNSC, head and neck squamous cell carcinoma; KIRC, clear cell renal cell carcinoma (ccRCC); LAML, acute myeloid leukemia; LUAD, lung adenocarcinoma; LUSC, lung squamous; OV, serous ovarian cancer; READ, rectal cancer; UCEC, endometrial cancer.

For this review, we surveyed the expression of genes related to Krebs cycle and Electron Transport Chain (ETC), in an RNA-seq dataset of 3,564 cancers representing 12 different cancer types in addition to ChRCC and representing a range of tissues of origin. The resulting heat map is shown in Figure 1, where sample profiles were clustered on the basis of selected metabolism-related genes. We find that the samples tend to segregate on the basis of high or low average expression of these genes, though for some tumor types, two or more subgroups may be defined. In particular, the ChRCC (“KICH”) cases form a tight and distinctive group, with the highest average expression of Krebs cycle and ETC genes relative to that of other tumor types. In contrast, ccRCC cases associate with multiple subgroups, on the basis of low to medium expression of Krebs cycle and ETC genes. From TCGA's previous ccRCC study, we know that the ccRCC cases with the lowest expression tend to be associated with worse patient outcome [16].

Additional pan-cancer studies to elucidate the role of metabolism and mitochondrial function in cancer could be undertaken, using genomic data resources from TCGA and elsewhere. While the LR-PCR method to sequence mtDNA in a large number of cancers could be fairly labor intensive, in our study we had the opportunity to compare mtDNA mutation results using either LR-PCR-based or WGS-based approaches [35], and we found these two to be highly complementary to each other. As the number of WGS profiles represented in TCGA is currently upwards of ~1,000 tumors [38], these data could be further mined for mtDNA mutations across various cancer types. One recent study by Larman *et al*. [35], surveyed mtDNA mutations in 226 tumors representing five types of cancer (colon adenocarcinoma, rectal adenocarcinoma, acute myeloid leukemia, glioblastoma, and ovarian serous cystadenocarcinoma) using WGS data, where 65% of somatic truncating mutations occurred in *MT-ND5*, suggesting these alterations affecting electron transport chain may not be limited to one rare tumor type. As a future set of studies into metabolism in cancer, metabolomic profiling data could be generated, in addition to our using mRNA expression as a surrogate for metabolite levels. In addition, more functional studies into the role of mtDNA mutations are needed, examining their effects on several cancer types, where we might consider alternative roles for complex I alteration in addition to the initiation of a Warburg effect as observed in some cancers [39].

### *TERT* promoter alterations in ChRCC

A highlight of our ChRCC study was the use of high-coverage WGS data covering the somatic and germline genomes (involving n=50 ChRCC cases) to uncover structural rearrangements involving the *TERT* promoter region. This finding underscores the value of taking a comprehensive genomic approach to a particular cancer, where unanticipated discoveries may arise from any one avenue of investigation. We first noted that three our cases manifested strong patterns of kataegis, a phenomenon involving highly localized substitution mutations (C>T or C>G) [12, 40]. The fact that this pattern was found in only a fraction of our cases invited us to compare ChRCC cases with and without kataegis. From RNA-seq analysis, the top most differentially expressed gene was *TERT*, which showed very high levels in kataegis cases as well as in a handful of additional cases. The question arose as to how *TERT* could be highly expressed in only a subset of ChRCC, while being at very low or undetectable levels in most of the other cases. The cases with the highest *TERT* expression did not have the previously described activating promoter mutations (C228T and C250T) and did not have copy gains to the extent of being able to explain the corresponding increase in expression. However, an examination of copy number levels in the *TERT* promoter region identified abrupt changes in copy for six ChRCC cases, which was indicative of structural breakpoints. These breakpoints were first confirmed by WGS analysis using Meerkat algorithm [41], and then independently validated by PCR.

*TERT* encodes a rate-limiting catalytic subunit of telomerase that maintains genomic integrity. Over 90% of cancers show an up-regulation of the telomerase enzyme, which can occur by *TERT* over-expression as well as by other means [3]. Previously known mechanisms for TERT up-regulation include point mutations in the promoter [2, 3], viral genome integration [42], gene amplification [43, 44], and germline polymorphisms [45]. The promoter mutations C228T and C250T, now observed in a wide variety of cancers (including bladder cancer, glioma, melanoma, squamous cell carcinoma, liver cancer, ovarian cancer, and thyroid cancer) [3, 38, 46], create de novo Ets/TCF binding sites, which can increase transcriptional activity from the promoter.

**Figure 2:**
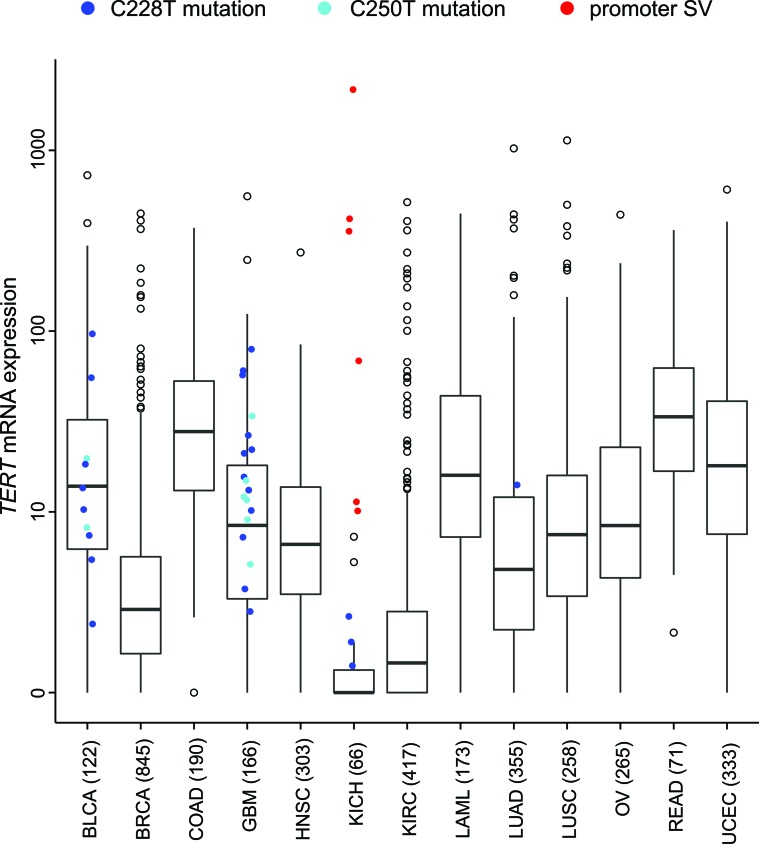
In a panel of human cancers of various tissues of origin (from TCGA, n = 3,564 samples), *TERT* mRNA expression levels by cancer type Also indicated are those cases previously found to harbor a TERT promoter mutation (C228T or C250T) by WGS analysis (n=555 human tumors)[1, 46], as well as the ChRCC (KICH) cases for which a structural variant (SV) breakpoint within the *TERT* promoter region was found [1]. For Box plots, boxes represent 25% (Q1), median, and 75% (Q3), and the upper whisker is the most extreme value that is no more than Q3+1.5 IQR and the lower whisker is the most extreme value that is no less than Q1-1.5 IQR. See Figure 1 legend for tumor type designations.

In this review, we surveyed *TERT* expression levels across >3,500 human cancers [20], including ChRCC (Figure 2). As with ChRCC, *TERT* mRNA levels vary widely within other cancer types, from undetectable to thousands of units by RNA-seq. Interestingly, for ChRCC, the somatically altered *TERT* subset defined the group of tumors displaying elevated TERT expression, with the majority of remaining tumors displaying quite low levels. For a subset of the cancers in Figure 2, WGS data were available, allowing us to note which cases were previously found to harbor the C228T or C250T mutations [46]. For the cancers surveyed here, *TERT* promoter mutations were mainly found within bladder cancers and glioblastomas, as well as for three of our ChRCC cases. While the point mutations tend to associate with higher *TERT* expression, most of the six ChRCC cases with promoter-associated structural variants (SVs) appear even higher than most cancers with the mutation. For most of the tumor cases outside of ChRCC represented in Figure 2, WGS data are not available, thus the status of *TERT* promoter alteration is currently unknown. To date, only in ChRCC cases have promoter SVs been thus far identified, and a survey of other cancer types for structural variants impacting *TERT* remains to be carried out.

To the best of our knowledge, the only instance of this phenomenon of *TERT* promoter SVs being observed previous to our study, was in a report by Zhao *et al*. [47] of non-tumorigenic, cultured fibroblast cells undergoing immortalization, which results provides some relevant context for our own findings. Zhao *et al*. studied *TERT* activation using genetically related pairs of telomerase-negative (Tel(−)) and -positive (Tel(+)) fibroblast lines. *TERT* was found translocated in all three Tel(+) cell lines examined but not in their parental pre-crisis cells and Tel(−) immortal siblings, with the breakage points being mapped to regions upstream of the *TERT* promoter. In light of the Zhao study, the observed *TERT* promoter rearrangements in our ChRCC cases may result from genomic instability in precancerous cells undergoing the crisis stage of immortalization, leading to activated telomerase. This hypothesis would be consistent with our ChRCC WGS analysis examining intra-tumor heterogeneity, where in most cases the *TERT* promoter SVs were estimated to reside in nearly all of the tumor cells, indicating that these rearrangements represent early events in the cancer.

While the *TERT* promoter-associated SVs were found with high recurrence in ChRCC and strongly associated with up-regulation of the gene, the mechanism of how this up-regulation occurs remains to be elucidated. In our ChRCC study, we carried out a systematic search for any cis-regulatory elements that might be commonly involved with the observed *TERT* promoter-associated SVs. In many cases, we did observe a number of cis-regulatory elements being placed in close proximity to the core promoter of *TERT*, as a result of rearrangement, although these results as yet do not lead us to a precise mechanism. Also, in the Zhao study [47], the authors had put forth the idea that *TERT* is normally silenced in non-cancerous cells, but that rearrangements upstream of *TERT* may allow the promoter to escape the repressive chromatin environment and thereby activate telomerase expression; we examined our TCGA DNA methylation data for any evidence of this in ChRCC. From our data, however, it was not clear that *TERT* is usually silenced in normal tissues, and we did not observe noticeable differences between the *TERT*-rearranged ChRCC samples and the rest. The caveat here is that our HM450 platform may not have the resolution to fully study the DNA methylation pattern around break points, as many of the involved regions are intergenic and have no coverage.

### Perspectives and Future Work: Pan-cancer analyses beyond the exome

To date, most large scale sequencing studies in cancer have largely focused on mutations within the exome [48, 49], which comprises on the order of 1% of the genome. Our ChRCC study demonstrates the utility of searching for potential driver alterations outside of the exome, e.g. through WGS and mtDNA profiling. For genomic datasets generated as part of efforts by TCGA and by the International Cancer Genomics Consortium (ICGC) [48], a large number of samples now have WGS data available, which data could and should be more deeply mined. A recent study by Weinhold *et al*. [38] analyzed WGS data from 863 human cancers of various types (most of these being from TCGA), in order to systematically identify noncoding regions that were recurrently mutated, revealing several novel regulatory changes. Future pan-cancer studies of whole genomes could and should include analysis of structural variants, which can have major effects on gene expression. Further, the analysis of mtDNA mutations lends further support for studies exploring this often overlooked segment of the genome. Our comprehensive molecular datasets on ChRCC should continue to serve as a resource for comparisons with future pan-cancer studies, or as a source for new areas to explore in cancer biology as well as for setting the stage for further investigations that lead to precision therapy directed toward the treatment of ChRCC.

## Abbreviations

ChRCC: chromophobe renal cell carcinoma
ccRCC: clear cell renal cell carcinoma
TCGA: The Cancer Genome Atlas
RNA-seq: RNA sequencing
mtDNA: mitochondrial DNA
WGS: whole genome sequencing
